# Type III CI factors from *w*AlbB induce cytoplasmic incompatibility via a Host Modification mechanism

**DOI:** 10.1371/journal.ppat.1014760

**Published:** 2026-10-29

**Authors:** Xinjun Hou, Feng Guang Goh, Sen Liu, Yao Liu, Xin Ee Yong, Qingsong Lin, Yu Cai

**Affiliations:** 1 Temasek Life Sciences Laboratory, National University of Singapore, Singapore; 2 Department of Biological Sciences, National University of Singapore, Singapore; Cornell University, UNITED STATES OF AMERICA

## Abstract

The widespread prevalence of the endosymbiotic bacterium *Wolbachia* among insect species relies on its ability to invade and manipulate host reproduction via several ways, among which cytoplasmic incompatibility (CI) is most studied. Two non-mutually exclusive models, the Host-Modification (HM) and the Toxin-Antidote (TA), have been proposed to explain the molecular basis of CI. Here, we investigate the CI-inducing activity of the Type III *CI* factors (*cifs*) from *w*AlbB, a strain currently being deployed to combat mosquito-borne diseases. This set of Cifs displays conserved subcellular localization dynamics in various insect cells. Both *cifA* and *cifB* are required for the induction of DNA damage in cultured cells, and transgenic *Drosophila*. Moreover, these *cifs* induce CI in *Drosophila* through the two-by-one manner, meaning *cifA* and *cifB* act together during spermatogenesis to induce CI, and maternal *cifA* can reverse the effect. Mechanistically, genetic, biochemical, cellular, and proteomic analyses reveal that this pair of *cifs* acts during the early stage of spermatogenesis but is dispensable in late-stage germline cells for CI induction. Collectively, our findings support the notion that *w*AlbB Type III *cifs* induce CI via a HM-based mechanism. Additional analyses suggest that different *Wolbachia* strains may employ distinct molecular mechanisms to induce CI.

## Author summary

Mosquito-borne diseases remain a major global health challenge, infecting hundreds of millions of people and causing hundreds of thousands of deaths each year. *Wolbachia*-based vector control is a promising strategy for reducing transmission of these pathogens and is currently being implemented in dozens of countries. This approach relies on cytoplasmic incompatibility (CI), a form of reproductive manipulation imposed by *Wolbachia*. Although the genes responsible for CI, known as *CI factors* (*cifs*), have been identified, the molecular mechanism underlying CI has remained controversial. We show here that Type III *cifs* from *w*AlbB induce CI through a Host Modification mechanism. We further provide evidence that different *Wolbachia* strains may use distinct molecular mechanisms to induce CI, with important implications for understanding and improving *Wolbachia*-based disease control strategies.

## Introduction

*Aedes aegypti* mosquitoes transmit several disease-causing pathogens, including dengue, Zika, and chikungunya viruses, posing a significant threat to human health [1,2]. Among current control strategies, the endosymbiotic bacterium *Wolbachia*-based vector control methods - implemented for population replacement (leveraging *Wolbachia*’s antiviral activity to reduce mosquito vector competence) and population suppression (taking advantage of *Wolbachia*-induced cytoplasmic incompatibility, CI, by releasing incompatible males) - stand out as promising approaches to mitigate disease transmission [3–6].

*Wolbachia pipientis* is an obligate intracellular bacterium that infects approximately 40–60% of insect species [7,8]. The widespread prevalence of *Wolbachia* infection stems from its ability to invade host populations and manipulate reproduction, thereby biasing its own maternal transmission through mechanisms such as male-killing, parthenogenesis, feminization, and cytoplasmic incompatibility (CI). Among these, CI is the most extensively studied [5,9]. CI occurs when *Wolbachia*-infected males mate with uninfected females, resulting in embryonic lethality and consequently, no viable offspring. However, this embryonic lethality can be rescued when females are infected with the same or a compatible *Wolbachia* strain [6,10–12].

Although the CI phenomenon was first described more than 50 years ago [13–15], the underlying genetic basis remained long elusive except for the proposed framework of modification (occurring in the sperm during spermatogenesis) and rescue (occurring in the fertilized egg to counteract the modification) [9] until the recent identification of the causative genes, the *CI* factors (*cifs*). *cifs* form an operon-like, syntenic two-gene pair - *cifA* (upstream) and *cifB* (downstream) - located within the Eukaryotic Association Module of prophage WO. At least one functional *cifA-cifB* pair has been identified in all CI-inducing *Wolbachia* strains, but not in non-CI-inducing strains [16–18]. Comparative genomic analyses have grouped *cif* genes into five clades (Types I-V) [12,18]. The CI-inducing properties of most *cifs* types (except Type V) have been validated using heterologous expression systems, including yeast, insect cell lines, and the insect model *Drosophila melanogaster* [16,17,19–21].

While the CI-inducing activities of *cifs* are well-documented, the underlying molecular mechanisms remain unsolved [22–24]. Two non-mutually exclusive models have been proposed, namely the Host-Modification (HM) model and the Toxin-Antidote (TA) model. The HM model, which originated from the modification/rescue framework and encompasses the titration-restitution, the mistiming, and the goalkeeper submodels, proposes that Cif proteins act during spermatogenesis (the pre-fertilization step) to modify sperm in such a way that the modified sperm product induces embryonic death upon fertilization, which can be rescuedby the action of the maternally deposited CifA. In this model, rescue does not require CifA-CifB interaction. A strong physical association between these cognate *cif* pairs has been demonstrated *in vitro,* supporting the notion that binding of CifA to CifB is likely required for CI induction, and these Cifs function via a two-by-one model to induce CI [9,21,25,26]. Conversely, the TA model posits that CifB acts as a toxin delivered with the sperm to the embryo, inducing embryonic lethality unless maternally deposited CifA neutralizes its toxin activity through direct binding [10,22,27]. Thus, the HM model proposes that CI occurs in the pre-fertilization stage during spermatogenesis, while the TA model implicates CI as post-fertilization events in the embryo. However, experimental evidence supporting one model does not necessarily exclude the other model [19,21,28–30].

Two *Wolbachia* strains have been widely deployed to combat mosquito-borne diseases, including *w*Mel from *Drosophila melanogaster* and *w*AlbB from *Aedes albopictus*. Releases of *Aedes aegypti* carrying either strain have significantly reduced the incidence of mosquito-borne diseases in targeted release areas [31–37]. The *w*AlbB genome encodes two distinct *cif* clades, a Type III and a Type IV [16,17]. The CI-inducing ability of Type IV *cif* genes has recently been confirmed in a transgenic *Aedes aegypti* system, supporting the TA mode for its CI mechanism [38].

In this study, we investigated the CI-inducing activity of the Type III *cif* genes (*cifA*_wAlbB[TIII]_ and *cifB*_wAlbB[TIII]_, also referred to as *cifA*^wAlbB^ and *cifB*^wAlbB^ by an alternative nomenclature [22]) from *w*AlbB and examined their functional mode. In this study, “*cifs*” refers collectively to *cifA* and *cifB*. Our results show that this Type III *cif* pair confers CI-inducing activity in a heterologous system. Cellular, genetic, biochemical, and proteomic evidence collectively support that this pair of *cifs* induces CI via a HM model rather than a TA model.

## Results

### Conserved subcellular localization of Cifs_wAlbB[TIII]_ in Drosophila and Mosquito cultured cells

*Drosophila* cultured cells is a heterologous system that has been utilized to investigate the subcellular localization of Cifs_wPip[TI]_, yielding results consistent with those obtained from *in vivo* transgenic *Drosophila* [19]. Here, we employed *Drosophila S2* cells to examine the subcellular localization of Cifs_wAlbB[TIII]_ and included Cifs_wPip[TI]_ as a reference. To this end, we cloned *cifs*_wAlbB[TIII]_ and *cifs*_wPip[TI]_ into an insect cell expressing vector driven by *Drosophila* ubiquitously expressed *actin5C* promoter (Fig 1A and S1A Fig).

**Fig 1:**
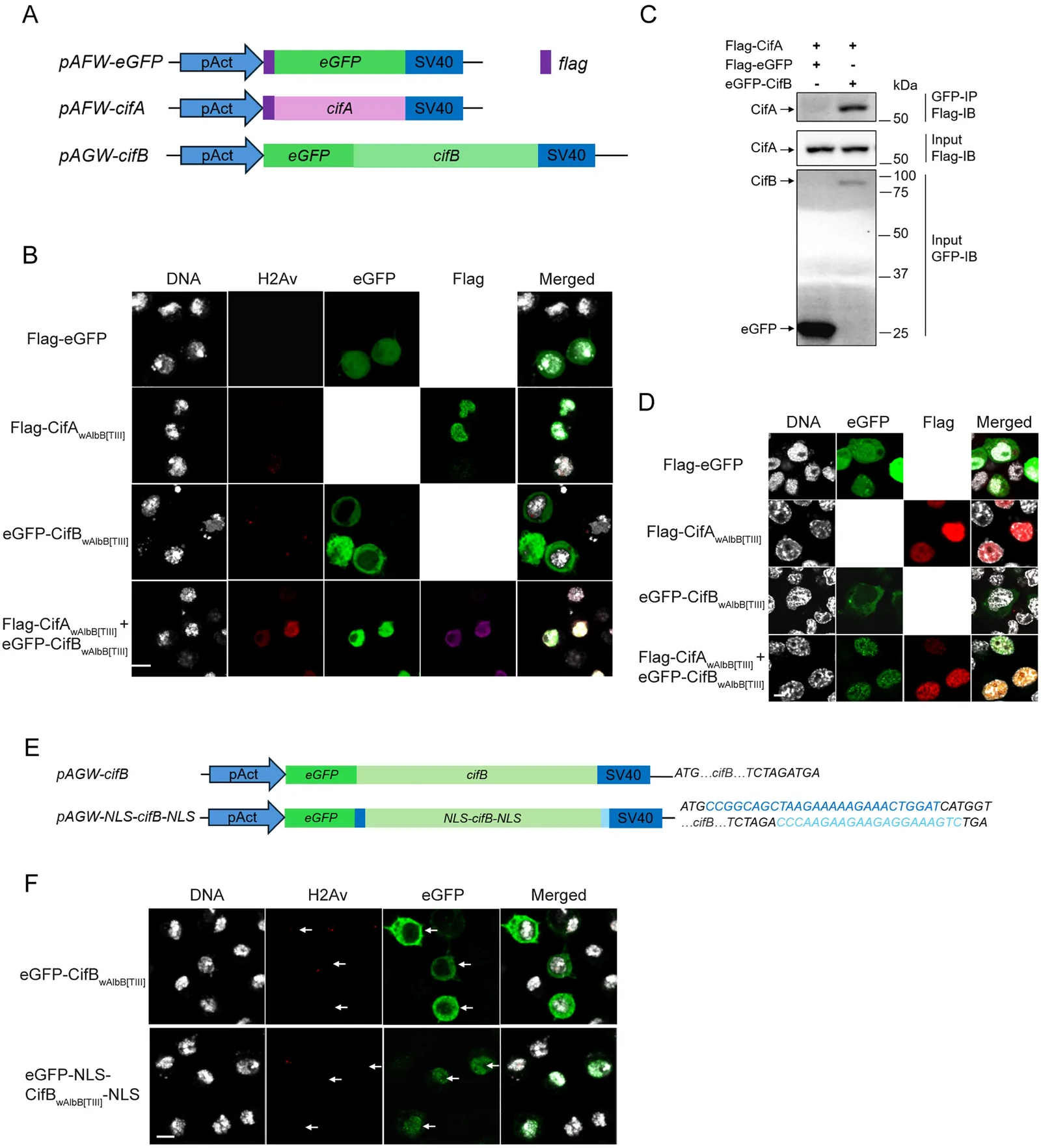
Dynamic Cifs_wAlbB[TIII]_ subcellular localization in *S2* and *C6/36* cells. A. Schematic diagram of *cifA*_wAlbB[TIII]_ and *cifB*_wAlbB[TIII]_ constructs. B. Representative images of Cifs_wAlbB[TIII]_ localization and p-γ-H2Av antibody expression in *S2* cells. C. When co-expressed in *S2* cells, Flag-CifA_wAlbB[TIII]_ can be pulled down by GFP-CifB_wAlbB[TIII]_ (right lane) but not Flag-eGFP (left lane). D. Representative images of Cifs_wAlbB[TIII]_ localization in *C6/36* cells. E. Schematic diagram of nuclear localization form of *cifB*_wAlbB[TIII]_ construct. F. Representative images of subcellular localization and p-γ-H2Av antibody expression of NLS-*cifB*_wAlbB[TIII]_-NLS in *S2* cells. Scale bar, 5 µm in B, D, and F.

When singly expressed in *S2* cells, CifA_wAlbB[TIII]_ was observed in the nucleus in the interphase cells, consistent with a putative nuclear localization signal (NLS) sequence “PSKRSR” present at its N-terminus (Fig 1B). Interestingly, CifB_wAlbB[TIII]_ alone exhibited cytoplasmic localization (Fig 1B). Notably, when co-expressed, both CifA_wAlbB[TIII]_ and CifB_wAlbB[TIII]_ localized to the nucleus (Fig 1B). Supporting the notion that cognate pair of Cifs interacts with each other, CifA_wAlbB[TIII]_ associated with CifB_wAlbB[TIII]_ in cultured cells (Fig 1C). These results show that CifA_wAlbB[TIII]_ influences the subcellular localization of CifB_wAlbB[TIII]_ in *Drosophila* cells. In contrast and as reported previously [19], when singly expressed, CifA_wPip[TI]_ was detected in the cytoplasm, whereas CifB_wPip[TI]_ localized in the nucleus in the interphase cells (S1B Fig). Co-expression of *cifA*_wPip[TI]_ and *cifB*_wPip[TI]_ resulted in the cytoplasmic localization of both CifA_wPip[TI]_ and CifB_wPip[TI]_ (S1B Fig). These data show that Cifs_wAlbB[TIII]_ and Cifs_wPip[TI]_ exhibited distinct subcellular localization in cultured cells.

We next examined their subcellular localization in cultured mosquito cells. Nuclear localization of CifA_wAlbB[TIII]_ and cytoplasmic localization of CifB_wAlbB[TIII]_ were detected when singly expressed in *C6/36* (derived from *Ae. albopictus*, Fig 1D) or *Aag2* (derived from *Ae. aegypti*) cells (S1C Fig). Similar to that observed in *S2* cells, co-expression of CifA_wAlbB[TIII]_ and CifB_wAlbB[TIII]_ led to their nuclear localization in these mosquito cells (Fig 1D and S1C Fig).

Together, these data demonstrate the conserved localization of Cifs_wAlbB[TIII]_ across different insect cell systems. These results are consistent with the notion that *Wolbachia* strains can exert their CI activity across a wide range of hosts [39–41].

### Cifs_wAlbB[TIII]_ acts together to induce DNA damage

All CifB proteins with CI activity contain two PD-(D/E)xK nuclease domains [16–18], and several of them have been shown to exhibit DNase activity *in vitro* or *in vivo* [16,20,28,38,42]. We went on to examine whether *cifs* expression in cultured cells could induce DNA damage, using the well-established marker phosphorylated-γ-H2Av (p-γ-H2Av) in *Drosophila*, which recognizes DNA double-strand breaks [43].

When *cifA*_wAlbB[TIII]_ was singly expressed in *S2* cells, no clear elevated p-γ-H2Av signal was detected, compared to untransfected controls (Fig 1B). Similarly, single *cifB*_wAlbB[TIII]_ expression did not upregulate p-γ-H2Av levels (Fig 1B). Interestingly, *S2* cells co-expressing *cifA*_wAlbB[TIII]_ and *cifB*_wAlbB[TIII]_ exhibited a clear p-γ-H2Av signal upregulation (Fig 1B), indicating elevated DNA double-strand breaks.

We then investigated the DNA damage upon *cifs*_wPip[TI]_ expression. While *cifA*_wPip[TI]_ was singly expressed in *S2* cells, no increase in the p-γ-H2Av signal was detected (S1B Fig). In contrast, single expression of *cifB*_wPip[TI]_ strongly elevated p-γ-H2Av levels compared with control cells (S1B Fig). Notably, co-expression of *cifA*_wPip[TI]_ suppressed the *cifB*_wPip[TI]_-induced elevated p-γ-H2Av signals (S1B Fig). These results are consistent with the subcellular localization of these Cifs_wPip[TI]_ that CifA_wPip[TI]_ directs cytoplasmic localization of CifB_wPip[TI]_ (S1B Fig), consistent with a previous publication [19]) and further confirm that this cell-based assay provides an informative system for dissecting Cifs’ activity.

Given that CifB_wAlbB[TIII]_ exhibits DNase activity and its nuclear localization is directed by CifA_wAlbB[TIII]_, these results suggest that CifA_wAlbB[TIII]_ mediates the nuclear localization of CifB_wAlbB[TIII]_, where CifB_wAlbB[TIII]_ executes its nuclease activity. Together, these results suggest that CifA_wAlbB[TIII]_ facilitates the nuclear localization of CifB_wAlbB[TIII]_ to induce DNA damage.

To test whether nuclear localization of CifB_wAlbB[TIII]_ alone is sufficient to induce DNA damage, we generated a CifB_wAlbB[TIII]_ variant fused with two nuclear localization signal (NLS) peptides and expressed it in *S2* cells (Fig 1E). As expected, this variant localized to the nucleus. It, however, did not induce elevated p-γ-H2Av signals (Fig 1F). These findings demonstrate that CifA_wAlbB[TIII]_ is essential for the CifB_wAlbB[TIII]_-mediated DNA damage.

### Cifs_wAlbB[TIII]_ induces CI via a two-by-one genetic model in transgenic *Drosophila*

We next employed the transgenic *Drosophila* model to determine whether *cifA*_wAlbB[TIII]_ and *cifB*_wAlbB[TIII]_ function similarly *in vivo* to induce DNA damage. Both *cifA*_wAlbB[TIII]_ and *cifB*_wAlbB[TIII]_ were cloned into the germline-optimized pUASP expression vector (Fig 2A) [44]. These constructs were injected into a *Wolbachia*-free *Drosophila* strain to establish transgenic lines. The UAS/Gal4 bipartite expression system was then used to drive ectopic expression of these transgenes in the testis [45].

**Fig 2:**
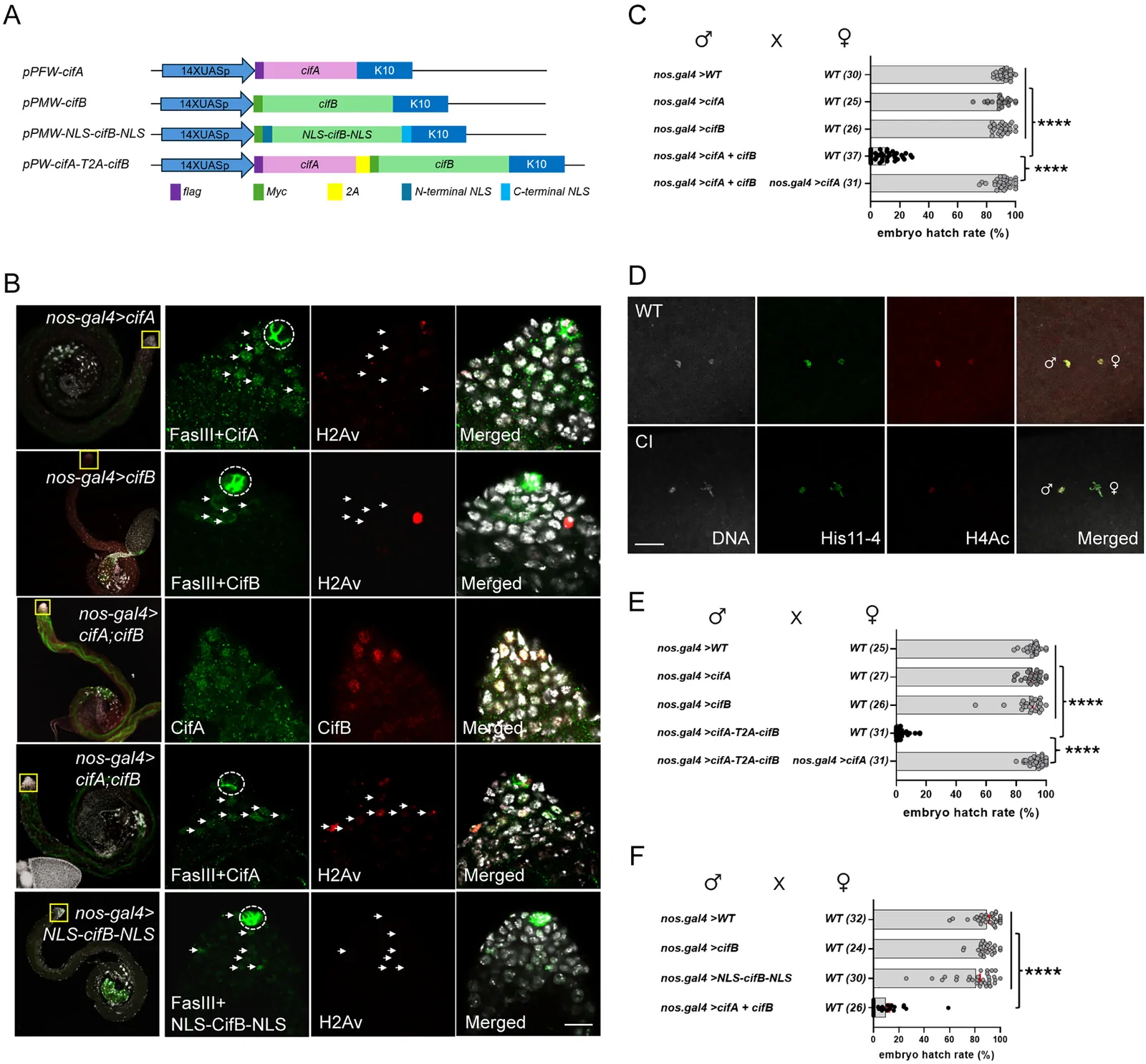
Cifs_wAlbB[TIII]_ induce CI in transgenic *Drosophila.* A. Schematic diagram of transgenic constructs. B. Localization of ectopically overexpressed Cifs_wAlbB[TIII]_ and p-γ-H2Av staining in *Drosophila* testis using the pan germline expression driver *nos-gal4*. The left panels are the overview of the testes, the zoom-in view anterior tip region (indicated by yellow box) are shown on the right panels. Scale bar, 10 µm. C. Embryo hatch rate analyses (by counting viable eggs) showing co-expression of *cifA*_wAlbB[TIII]_ and *cifB*_wAlbB[TIII]_ in testes induces CI, which is rescued by female expressing *cifA*_wAlbB[TIII]_ only. Each circle represents the percent of embryos that hatched from a single male and female pair. Total number was listed in parentheses. Significant differences were determined by a Kruskal–Wallis test and Dunn multiple comparison tests. D. Synchronous chromosome condensation of male pronucleus (high H4.Ac signal) and female pronucleus (low H4.Ac signal) are observed in WT control embryos. CI arrested embryos show asynchronous chromosome condensation. Scale bar, 20 µm. E. Embryo hatch rate analyses showing that expression of CifA-T2A-CifB induces CI, which can be rescued by female expressing *cifA*_wAlbB[TIII]_. F. Hatch rate analyses showing that single expression of NLS- *cifB*_wAlbB[TIII]_-NLS does not induce CI.

When driven by the strong pan-germline cell driver *nos-gal4* [46], singly expressed CifA_wAlbB[TIII]_ was detected in anterior germline cells, including germline stem cells (GSCs), gonioolbasts (GBs) and early spermatogonia, with a nuclear localization (Fig 2B, white arrows), reminiscent of its localization in the cultured cells (Fig 1B). These CifA_wAlbB[TIII]_-expressing cells did not exhibit clear elevated p-γ-H2Av signals (Fig 2B). CifB_wAlbB[TIII]_, when singly expressed, was also observed in the germ cells near the tip, where it localized to the cytoplasm (Fig 2B), consistent with its localization in the cultured cells. No elevated p-γ-H2Av signal was detected in these cells either (Fig 2B).

Interestingly, when co-expressed, CifA_wAlbB[TIII]_ and CifB_wAlbB[TIII]_ co-localized in the nucleus and induced elevated p-γ-H2Av signals in these cells (Fig 2B). Furthermore, a CifB_wAlbB[TIII]_ variant tagged with NLS exhibited nuclear localization but failed to induce p-γ-H2Av upregulation (Fig 2B). Together, these data indicate that CifA_wAlbB[TIII]_ and CifB_wAlbB[TIII]_ function together to induce DNA damage upon ectopic expression in *Drosophila* male germline cells.

*w*AlbB genome encodes two sets of CI factors, *cifs*_wAlbB[TIII]_ and *cifs*_wAlbB[TIV]_. While the CI activity of *cifs*_wAlbB[TIV]_ has recently been verified [38], the CI-inducing activity of *cifs*_wAlbB[TIII]_ and its functional mode of *cifs*_wAlbB[TIII]_ remain untested. We therefore examined whether *cifs*_wAlbB[TIII]_ could induce CI in *Drosophila* by measuring the hatch rate of embryos, following established protocols [16,17]. Embryos derived from *Wolbachia*-free females that mated with males expressing either *cifA*_wAlbB[TIII]_ or *cifB*_wAlbB[TIII]_ driven by *nos-gal4* exhibited hatch rates comparable to control crosses (Fig 2C). In contrast, embryos of *Wolbachia*-free females that mated with males co-expressing *cifA*_wAlbB[TIII]_ and *cifB*_wAlbB[TIII]_ showed a markedly reduced hatch rate (Fig 2C). Cytological examination revealed that early embryos exhibited asynchronized maternal and paternal chromosome condensation, a hallmark of *Wolbachia*-induced CI (Fig 2D) and in line with a previous publication [25]. Importantly, this reduced hatch rate was fully reverted to control levels when females expressing *cifA*_wAlbB[TIII]_ were used in the crosses (Fig 2C), confirming the CI-inducing activity of *cifs*_wAlbB[TIII]_.

To confirm this, we generated additional transgenes carrying an operon-mimicking construct in which *cifA*_wAlbB[TIII]_ and *cifB*_wAlbB[TIII]_ were linked by a T2A sequence (*cifA-T2A-cifB*), which causes ribosomal skipping during translation and leads to the production of two separate CifA_wAlbB[TIII]_ and CifB_wAlbB[TIII]_ polypeptides and established such transgenes (Fig 2A). Similarly, males expressing *cifA-T2A-cifB* caused a strong reduction in hatch rate when mated with *Wolbachia*-free females, which was reverted to control levels when mated with females expressing *cifA*_wAlbB[TIII]_ (Fig 2E). While the NLS-tagged CifB_wAlbB[TIII]_ variant localized in the nucleus, it did not induce CI when ectopically expressed in the testes (Fig 2F), supporting the role of CifA_wAlbB[TIII]_ in CI induction.

Together, these results demonstrate that the Type III CI factors of *w*AlbB act through a two-by-one genetic model to induce CI in a heterologous *Drosophila* model [21,47].

### Cifs_wAlbB[TIII]_ acts in early germline cells to induce CI

We next investigated the mechanism underlying CI induced by *cifs*_wAlbB[TIII]_, taking advantage of the extensive genetic tools available in *Drosophila*, which allow precise temporal and spatial control of gene expression.

The *nos-gal4* driver is active in GSCs, GBs, and all spermatogonia but low in spermatocytes (Fig 3A and S2 Fig) [46,48]. To determine which of these cell populations is responsible for the observed CI phenotype, we expressed *cifs*_wAlbB[TIII]_ (either a combination of *cifA*_wAlbB[TIII]_ + *cifB*_wAlbB[TIII]_ or *cifA-T2A-cifB*) using a panel of cell type-specific *gal4* drivers (Fig 3A).

**Fig 3:**
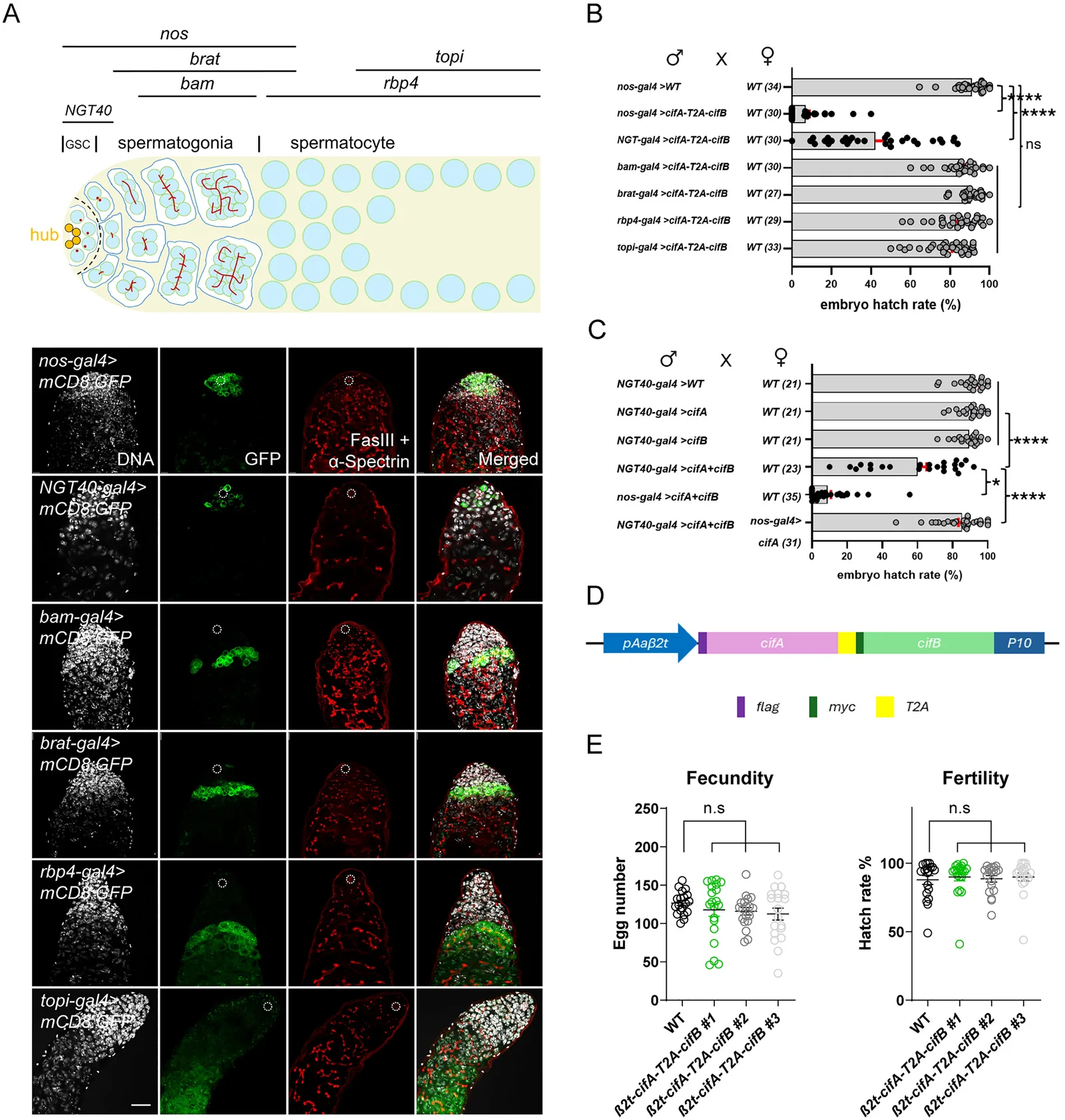
Cifs_wAlbB[TIII]_ function in early germline cells to induce CI. A. Top panel, schematic diagram of expression pattern of different *gal4* lines tested. Bottom panel, expression patterns of respective *gal4* lines crossed with *UAS-mCD8-GFP* reporter. Hoechst in white; GFP in Green; FasIII (a hub cell marker) and α-Spectrin (a fusome marker) in red. Dot circles indicate the hub cells. Scale bar: 20 µm. B. Embryo hatch rate assays showing ectopic expression of *cifs*_wAlbB[TIII]_ by *nos-gal4* and *NGT40-gal4* but not *bam-gal4*, *brat-gal4*, *rbp4-gal4* or *topi-gal4* induces CI. C. Embryo hatch rate results showing that co-expression of *cifA*_wAlbB[TIII]_ and *cifB*_wAlbB[TIII]_ but not their single expression by *NGT40-gal4* induces rescuable CI by maternal supplied *cifA*_wAlbB[TIII]_. D. Schematic diagram of mosquito transgenic construct *β2t*-flag-*cifA*_wAlbB[TIII]_-T2A-myc-*cifB*_wAlbB[TIII]_. E. Fecundity and fertility of *Aedes aegypti β2t*-flag-*cifA*_wAlbB[TIII]_-T2A-myc-*cifB*_wAlbB[TIII]_ transgenic lines. (Kruskal-Wallis test, *p*-value>0.05).

Surprisingly, expression of *cifs*_wAlbB[TIII]_ under *bam-gal4* (active in late spermatogonia [49]), *brat-gal4* (active in late spermatogonia and early spermatocytes [46]), *rpb4-gal4* (active in early and late spermatocytes [50]), or *topi-gal4* (active in late spermatocytes [51]) did not induce a CI phenotype (Fig 3B). This contrasts with previous findings showing that ectopic expression of *cifs*_wPip[TI]_ or *cifs*_wAlbB[TIV]_ under a late spermatocyte *topi* promoter induced a strong CI in *Drosophila* or *Aedes aegypti*, respectively [38]. These results suggest that early germline cell expression of *cifA*_wAlbB[TIII]_ and *cifB*_wAlbB[TIII]_ might be essential for their CI-inducing activity.

To test this, we screened a small collection of germline cell-expressing g*al4* drivers by crossing each driver to a *UAS-mCD8-GFP* responder line. Among them, we identified *NGT40-gal4*, which drives reporter expression in GSCs, GBs, early spermatogonia, but not late spermatogonia (Table 1 and Fig 3A), as previously reported [52]. Strikingly, males expressing *cifs*_wAlbB[TIII]_ under *NGT40-gal4* induced CI when crossed with *Wolbachia*-free females, which was fully rescued by maternal expression of *cifA*_wAlbB[TIII]_ (Fig 3C).

**Table 1:** List of *gal4* lines screened and their expression pattern in this study.

BDSC#	Genotype	Expression in testis
**4442**	y[1] w[*]; P{w[+mC]=GAL4-nanos.NGT}40	**Spermatogonia**
**7303**	*P{w[+mC]=GAL4::VP16-nanos.UTR}MVD2, w[1118]*	No expression
**25394**	w[*]; Pen[D14]/CyO; P{w[+mC]=GAL4-nanos.NGT}9/TM6B, Tb[+]	**Spermatogonia**
**38838**	*w[1118]; P{y[+t7.7] w[+mC]=GMR52F01-GAL4}attP2*	No expression
**38864**	*w[1118]; P{y[+t7.7] w[+mC]=GMR53B07-GAL4}attP2*	No expression
**38868**	*w[1118]; P{y[+t7.7] w[+mC]=GMR53C01-GAL4}attP2*	No expression
**38869**	*w[1118]; P{y[+t7.7] w[+mC]=GMR53C02-GAL4}attP2*	No expression
**41276**	*w[1118]; P{y[+t7.7] w[+mC]=GMR52F03-GAL4}attP2/TM3, Sb[1]*	No expression
**48192**	*w[1118]; P{y[+t7.7] w[+mC]=GMR52F05-GAL4}attP2*	No expression
**49581**	*w[1118]; P{y[+t7.7] w[+mC]=GMR52G12-GAL4}attP2*	No expression
**50433**	*w[1118]; P{y[+t7.7] w[+mC]=GMR53E04-GAL4}attP2*	No expression
**50448**	*w[1118]; P{y[+t7.7] w[+mC]=GMR53G06-GAL4}attP2*	No expression
**58424**	*P{w[+mC]=otu-GAL4::VP16.R}1, w[1118]*	No expression
**62770**	*w[1118] PBac{w[+mC]=IT-gal4}ovo[0305-G4]*	No expression

As documented in the early sections, the subcellular localization of Cifs_wAlbB[TIII]_ is conserved between *Drosophila* and *Aedes aegypti* cells, supporting a conserved mechanism. We therefore examined whether forced expression of *cifs*_wAlbB[TIII]_ in *Aedes* testes could also induce CI. For this, we placed the *T2A* version of *cifs*_wAlbB[TIII]_ (*cifA-T2A-cifB)* under a *beta-2-tubulin* (*β2t*) promoter [53], a testis-specific promoter that is strongly active in spermatocytes (Fig 3D), and generated transgenic *Aedes aegypti* lines using the *piggyBac* transgenic system [54,55]. Several independent lines were established. When transgenic males were crossed with uninfected females, no CI phenotype was observed, indicating that ectopic expression of *cifs*_wAlbB[TIII]_ in spermatocytes does not induce CI (Fig 3E). This finding differs from the previous report showing that expression of *cifs*_wAlbB[TIV]_ under the same promoter results in complete male sterility [38].

Collectively, these data show that, unlike *cifs*_wPip[TI]_ or *cifs*_wAlbB[TIV]_, forced expression of *cifs*_wAlbB[TIII]_ during late spermatogenesis (in late spermatogonia and spermatocytes) failed to induce CI. Instead, ectopic expression of *cifs*_wAlbB[TIII]_ in early germline cells (including GSCs and GBs) can induce CI.

### Cifs_wAlbB[TIII]_ acts through a HM model to induce CI

The CI-inducing activity of *cifs*_wAlbB[TIII]_ in the early but not late germline cells suggests that these CI factors act through a Host Modification (HM) model. To test this hypothesis, we examined the expression dynamics of Cifs_wAlbB[TIII]_ during spermatogenesis and included Cifs_wPip[TI]_ as a control.

As reported previously [19], when driven by *topi-gal4*, both CifA_wPip[TI]_ and CifB_wPip[TI]_ were detected in late spermatocytes and developing spermatids (S3A Fig). Similarly, CifA_wPip[TI]_ and CifB_wPip[TI]_ were observed in spermatocytes and spermatids when driven by *brat-gal4* driver (S3A Fig). Notably, when *cifs*_wPip[TI]_ were expressed under the early germline cell driver *NGT40-gal4*, CifB_wPip[TI]_ was also detected in spermatids (Fig 4A). These observations indicate that Cifs_wPip[TI]_ expressed in early germline cells is stable and persists into late-stage germline cells. This finding, consistent with previous reports showing that CifB_wPip[TI]_ is delivered to the embryo along with the sperm, supports the TA model of CI for this set of Cifs [19].

**Fig 4:**
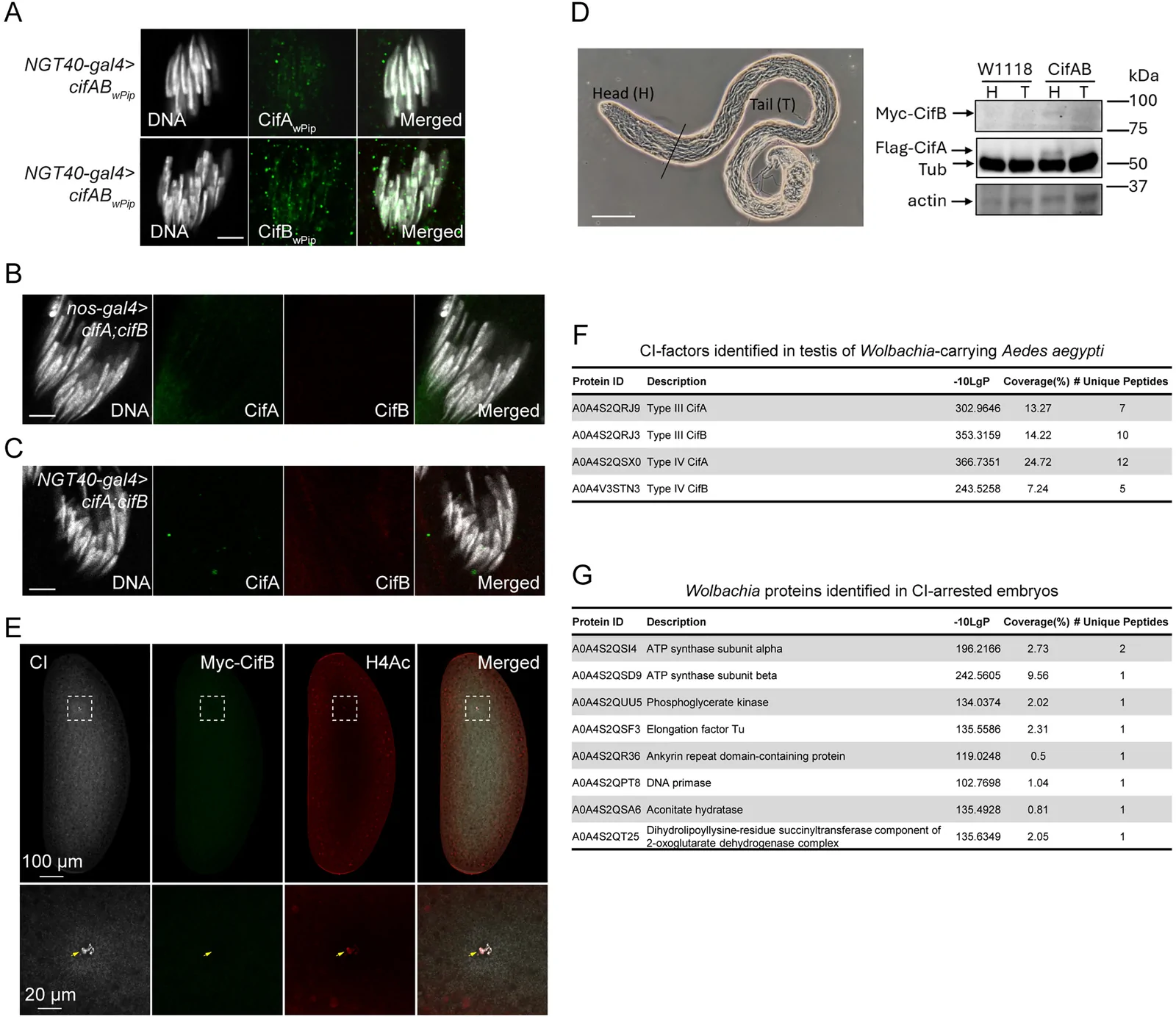
Cifs_wAlbB[TIII]_ not transferred to the fertilized embryos. A. CifA_wPip[TI]_ and CifB_wPip[TI]_, driven by *NGT40-gal4* that expresses in GSCs, GBs and early spermatogonia, are detected in spermatids. Scale bar, 5 µm. B and C. Neither CifA_wAlbB[TIII]_ nor CifB_wAlbB[TIII]_ driven by the pan germline cell driver (including GSCs, GBs, spermatogonia, and early spermatocytes) (B) or *NGT40-gal4* (C) is detected in spermatids, suggesting that these proteins are not loaded into mature sperms. Scale bar, 5 µm. D. Left: a wild-type testis under light microscope, black line indicates the position to bisect head (H) and tail (T) portion of Drosophila testes. Scale bar, 100 µm. Right: Western blot analysis of CifA_wAlbB[TIII]_ and CifB_wAlbB[TIII]_ presence in the H and T portion of control (*w*^*1118*^) and *Cifs*_wAlbB[TIII]_-expression testes driven by *nos-gal4*. E. CifB_wAlbB[TIII]_ is not detected on the H4.Ac-positive paternal chromatin in CI-arrested *Drosophila* embryos derived from uninfected females crossed with males carrying *nos-gal4* and *cifs*_wAlbB[TIII]_. F. Proteomic analyses show that both Cifs_wAlbB[TIII]_ and Cifs_wAlbB[TIV]_ are detected in *Aedes aegypti* testis infected with *w*AlbB. G. Eight *Wolbachia* polypeptides (without Cifs_wAlbB[TIII]_) are identified in CI-arrested embryos by proteomic analysis.

In contrast, when driven by *nos-gal4*, both CifA_wAlbB[TIII]_ and CifB_wAlbB[TIII]_ were detected in anterior germline cells (Fig 2B). Neither protein was observed in late-stage germline cells including spermatids (Fig 4B). Similarly, when driven by *NGT40-gal4*, CifA_wAlbB[TIII]_ or CifB_wAlbB[TIII]_ were not detected in spermatids despite their ability to induce CI (Fig 4C). The absence of detectable Cifs_wAlbB[TIII]_ in late-stage germline cells could be due to its rapid turnover, as *nos-gal4* is a strong pan-germline cell expression driver. To test this, we analysed Cifs_wAlbB[TIII]_ expression under the late spermatogonia (*brat-gal4*) or spermatocyte driver (*rbp4-gal4* and *topi-gal4*). Again, neither CifA_wAlbB[TIII]_ nor CifB_wAlbB[TIII]_ was detected in spermatids under any of these drivers (S3B Fig). Likewise, in *Aedes aegypti*, Cifs_wAlbB[TIII]_ were not detected in spermatids when expressed under the strong spermatocyte promoter *β2t* (S3C Fig). Together, these results show the absence of Cifs_wAlbB[TIII]_ in spermatids and indicate that, unlike Cifs_wPip[TI]_, Cifs_wAlbB[TIII]_ is likely not loaded into sperms.

To further assess whether Cifs_wAlbB[TIII]_ associates with sperms, we performed western blot analyses on dissected testes expressing *cifs*_wAlbB[TIII]_ under *nos-gal4*. Testes were bisected into two regions, an anterior portion containing germline cells ranging from GSCs to spermatids and a posterior portion enriched for mature spermatozoa (Fig 4D). As expected, both CifA_wAlbB[TIII]_ and CifB_wAlbB[TIII]_ were detected in samples derived from the anterior portion, consistent with their presence in early germline cells, neither CifA_wAlbB[TIII]_ nor CifB_wAlbB[TIII]_ was present in the sperm-containing posterior portion (Fig 4D). This further supports that Cifs_wAlbB[TIII]_ is not loaded with sperms and thus unlikely to be delivered to embryos.

We further examined whether CifB_wAlbB[TIII]_ could be delivered into the embryos. We took advantage of the CI-arrested embryos by examining the presence of CifB_wAlbB[TIII]_. Similar to a previous report [56], CI-arrested embryos exhibited abnormal Histone H4 deposit on parental chromatin (Fig 4E). In line with early data, CifB_wAlbB[TIII]_ was not detected on the parental chromosome, differing from the early report showing that CifB_wPip[TI]_ was present in CI-arrested embryos [19].

Finally, we conducted mass spectrometry analyses to test whether Cifs_wAlbB[TIII]_ are transferred to embryos. In *Drosophila*, embryogenesis occurs rapidly following sperm entry, and the first zygotic division occurs approximately 15 min after fertilization [57]. This rapid embryonic development makes it difficult to collect enough early samples for analysis. In contrast, in *Aedes aegypti*, the fusion of male and female pronuclei occurs by the end of 1 hr after sperm entry [58]. We turned to *w*AlbB-infected *Aedes aegypti* to examine the presence of CifB_wAlbB[TIII]_ in testes and CI-arrested embryos under its native condition without transgenic expression. As expected, both Cifs_wAlbB[TIII]_ and Cifs_wAlbB[TIV]_, were detected in the testes among about 450 *Wolbachia*-derived peptides, confirming infectious status (Fig 4F and S1 Table). Under our experimental conditions, only eight *Wolbachia*-derived polypeptides were detected in CI-arrested embryos collected within 20 min after egg laying contained, none of which corresponds to CifA_wAlbB[TIII]_ and CifB_wAlbB[TIII]_ (Fig 4G).

Together, these results demonstrate that Cifs_wAlbB[TIII]_ are expressed in early germline cells but are not transferred to fertilized embryos. These data strongly support the notion that Cifs_wAlbB[TIII]_ induces CI through a HM mechanism, acting during spermatogenesis rather than post-fertilization.

## Discussions

*Wolbachia* are most successful intracellular bacteria known to invade and spread across diverse insect species. To achieve this success, *Wolbachia* employ a wide range of reproductive manipulation strategies, including male-killing, parthenogenesis, feminization, and CI. Although CI has been extensively studied, the underlying molecular mechanism remains unsolved, with multiple hypotheses proposed that can be grouped into two general models: the host-modification (HM) and toxin-antidote (TA) models [11,12,27,59,60].

In this study, we investigated the CI-inducing activity and functional mechanism of the Type III *CI factors* of *w*AlbB. Our data show that Cifs_wAlbB[TIII]_ exhibited conserved subcellular behaviour across different heterologous systems, including cultured cells and transgenic *Drosophila*, and induces DNA damage. In *Drosophila*, co-expression of *cifA*_wAlbB[TIII]_ and *cifB*_wAlbB[TIII]_ in the testis induced a CI phenotype, which was rescued by females expressing *cifA*_wAlbB[TIII]_. Further analyses revealed that expression of *cifs*_wAlbB[TIII]_ in early, but not late, germline cells is essential for CI-induction. Consistent with this, Cifs_wAlbB[TIII]_ were detected in early but not late germline cells or CI-arrested embryos, suggesting that CifB_wAlbB[TIII]_ is not transmitted to embryos with sperms. Similarly, in *w*AlbB-infected mosquitoes, Cifs_wAlbB[TIII]_ was detected in the testis but absent in CI-arrested embryos. Collectively, these findings strongly support that *cifs*_wAlbB[TIII]_ induces CI via a HM, rather than a TA, model.

The key distinction between the HM model and the TA model lies in the site of action – that is, where the modification occurs, and where the rescue takes place. The HM model posits that Cifs modify sperm chromatin or other host factors during spermatogenesis, and Cifs need not be transferred to embryos, where the rescue occurs [29]. In contrast, the TA model proposes that CifB (the toxin) is delivered with sperm and acts in the fertilized embryo unless its toxicity is neutralized by maternally deposited CifA through direct binding to CifB[22, 38]. Although both models have received substantial experimental supports, evidence favoring one model does not necessarily exclude the other one [22–24].

For instance, one important piece of evidence for the TA model comes from analyses of the heterologous expression of *cifs*_wPip[TI]_ in *Drosophila* testes [19]. Both CifA_wPip[TI]_ and CifB_wPip[TI]_ were detected in early spermatids, but only CifB_wPip[TI]_ was observed in late spermatids and transmitted to fertilized embryos. In early embryos, CifB_wPip[TI]_ localized to paternal chromatin and was associated with DNA replication defects. These findings support the TA mode of action for this set of CI factors. However, it remains unclear whether Cifs_wPip[TI]_ expression in early germline cells induces modifications, such as DNA damage, which are transmitted to fertilized embryos and contribute to CI. It is worth noting that the background expression of *cifB*_wPip[TI]_ in this transgenic line (presumably in all germline cells, including GSCs, GBs, and spermatogonia) is sufficient to induce complete CI. Furthermore, no rescue data has been provided for the induced CI.

Another piece of evidence supporting the TA model derives from the analyses of *cifs*_wAlbB[TIV]_ in a transgenic *Aedes aegypti* model [38]. When *cifs*_wAlbB[TIV]_ was expressed under the strong spermatocyte *β2t* promoter, it resulted in em*b*ryonic lethality that is not rescued by *w*AlbB-carrying females. DNA breaks were also observed in these sperms, suggesting possible sperm modification. Expression of Cifs_wAlbB[TIV]_ under a weaker spermatocyte-specific (*topi*) promoter resulted in complete but rescuable CI without detectable DNA damage in sperms, lending support to the TA model. However, this study does not exclude the possibility that a low level of DNA damage, below the detection limit of the TUNEL staining, is induced and sufficient to induce CI. Moreover, all data were obtained under conditions of ectopic expression, and it is unclear whether the observed Cif expression levels are comparable to those in *Wolbachia*-infected testes. Thus, while these data strongly support the TA model, they cannot effectively rule out the possibility that sperm are modified before fertilization.

Conversely, data derived from *w*Mel in *Drosophila* provide strong support to the HM model of CI [29,61]. Both endogenous (*w*Mel-infected) and overexpressed CifA_wMel[TI]_ and CifB_wMel[TI]_ were detected in the germline cells throughout spermatogenesis. Interestingly, defective histone retention in elongating spermatids and protamine deficiency in mature sperms were observed in these testes. Furthermore, protamine deficiency was also detected in inseminated sperms in female spermathecae and seminal receptacle, but not in early fertilized embryos, lending strong support to the HM model of CI. Of note, both CifA_wMel[TI]_ and CifB_wMel[TI]_ were detected in mature sperms. It is worth noting that in *Drosophila melanogaster*, embryogenesis proceeds rapidly, with the first mitotic division occurring approximately 15 min post-fertilization [57,62]. It remains unclear whether Cifs_wMel[TI]_ transferred with sperms are removed before, during, or after fertilization, which occurs in the female reproductive tract before egg laying, as the molecular event leading to the asynchronous maternal and paternal chromosome behaviour in CI embryos remains unknown. Hence, these results also do not completely exclude the TA model of CI. Interestingly, a recent publication demonstrated that in *Drosophila*, CI-like phenotype could be induced by a small molecule targeting histone acetylation and reverted by *w*Mel-carrying females, providing the clear support that *cifs*_*w*Mel[TI]_ induces CI via the HM model [63]. It’d be interesting to test if other *cifs* function similarly.

Our results show in this study that when ectopically expressed in *Drosophila* testes by *nos-gal4* driver, both CifA_wAlbB[TIII]_ and CifB_wAlbB[TIII]_ were detected in GSCs and early spermatogonia, but not in spermatids, and mature sperms. Neither CifA_wAlbB[TIII]_ nor CifB_wAlbB[TIII]_, driven by spermatogonia (*bam-gal4* or *brat-gal4*) or spermatocyte (*rbp4-gal4* and *topi-gal4*) drivers, was detected in the late-stage spermatids and mature sperm cells. Similarly, in *Ae. aegypti*, CifA_wAlbB[TIII],_ and CifB_wAlbB[TIII]_ were not detected in sperms in the testes expressing *cifs*_wAlbB[TIII]_ under the control of the strong spermatocyte (*β2t*) promoter. These results indicate that CifA_wAlbB[TIII]_ and CifB_wAlbB[TIII]_ are likely not loaded into sperms. Importantly, expression of *cifs*_wAlbB[TIII]_ under these spermatogonia and spermatocyte drivers does not induce CI. In contrast, *cifs*_wAlbB[TIII]_ expression by an early germline driver *NGT40-gal4*, which expresses in GSCs, GBs and early spermatogonia, induced CI. These results demonstrate that expression of this *cif* pair in early, but not late, germline cells is essential for CI induction. Supporting this, Western blot analyses showed that CifA_wAlbB[TIII]_ and CifB_wAlbB_ were detected in samples derived from the anterior portion of the testes, including GSCs, GBs, and spermatogonia, but not in samples containing mature sperms. Furthermore, proteomic analysis revealed that, in *w*AlbB-infected mosquitoes, Cifs_wAlbB[TIII]_ were detected in the testes but not in fertilized (CI-arrested) embryos. While we cannot exclude the possible presence of low amounts of Cifs below the limit of these detection methods, these data collectively, support the notion that CifA_wAlbB[TIII]_ and/or CifB_wAlbB[TIII]_ are not transmitted along with sperms to fertilized embryos and strongly argue that *cifs*_wAlbB[TIII]_ acts through the HM rather than the TA model to induce CI.

Is there a unified CI-inducing mechanism shared by all functional Cif pairs? Results from this and previous studies suggest that different *cif* pairs may exert their CI effects via distinct molecular mechanisms. First, Cifs_wPip[TI]_ and Cifs_wAlbB[TIII]_ exhibit different subcellular dynamics. In cultured cells, CifA_wPip[TI]_ localizes to the cytoplasm, CifB_wPip[TI]_ shows nuclear localization, and CifA_wPip[TI]_, when co-expressed, induces cytoplasmic retention of CifB_wPip[TI]_ [19] (S1B Fig). Conversely, our results show CifA_wAlbB[TIII]_ localizes to the nucleus, and CifB_wAlbB[TIII]_ exhibits cytoplasmic localization. When co-expressed, both CifA_wAlbB[TIII]_ and CifB_wAlbB[TIII]_ localize to the nucleus (Fig 1B). Furthermore, a previous publication showed that endogenous CifA_wMel[TI]_ exhibits nuclear localization in *w*Mel-infected Drosophila testis. Second, our results show that while singly expressed CifB_wPip[TI]_ induces elevated DNA damage, CifA_wPip[TI]_ co-expression causes cytoplasmic retention of CifB_wPip[TI]_ and attenuates DNA damage (S1B Fig). In contrast, CifB_wAlbB[TIII]_ does not induce DNA damage when localized in the cytoplasm but promotes DNA damage when co-localized with CifA_wAlbB[TIII]_ in the nucleus (Fig 1B). Similar clear and elevated DNA damage was observed in the testes of transgenic *Drosophila* co-expressing *cifs*_wAlbB[TIII]_ but not testes with single expression of *cifA*_wAlbB[TIII]_ or *cifB*_wAlbB[TIII]_ (Fig 2B). Similarly, a previous study showed that co-expression of *cifs*_wMel[TI]_ in *Drosophila* male germline cells also induced strong DNA damage [42]. Third, consistent with these localization and DNA damage patterns, single expression of *cifA*_wAlbB[TIII]_ or *cifB*_wAlbB[TIII]_ does not induce CI, whereas their co-expression leads to a strong CI (Fig 2C). In contrast, earlier reports documented that expression of *cifB*_wNo[TIII]_ or *cifB*_wAlbB[TIV]_ alone induces a strong CI, which can be rescued by maternal supplied *cifA*_wNo[TIII]_ or *cifA*_wAlbB[TIV]_, respectively [28,30,38]. Fourth, our results show that when *cifs*_wPip[TI]_ is expressed under the early germline driver *NGT40-gal4*, Cifs_wPip[TI]_ are detected in late-stage spermatids (S3A Fig). Similarly, Cifs_wPip[TI]_ are loaded into sperm when driven by the spermatocyte driver *topi-gal4* [19]. In contrast, Cifs_wAlbB[TIII]_ are detected only in early germline cells (including GSCs and GBs) but not in late-stage spermatids under *nos-gal4* (Fig 2B and 4B). Neither CifA_wAlbB[TIII]_ nor CifB_wAlbB[TIII]_ is detected in spermatids under the spermatocyte drivers *rbp4-gal4* or *topi-gal4* (S3B Fig). These data strongly suggest that *cifs*_wPip[TI]_ likely induces the CI via the TA model, whereas *cifs*_wAlbB[TIII]_ functions through the HM model. Considering the great diversity of *Wolbachia* strains, it is likely that different strains may employ different molecular mechanisms to induce CI. Understanding these mechanisms will facilitate the rational design of CI-based vector control strategies.

## Materials and methods

### Ethics statement

This study was reviewed and approved by the Institutional Biosafety Committee (IBC) of Temasek Life Sciences Laboratory, Singapore. All procedures involving mosquitoes (IBC-031521-CY-0, GMAC Ref No.:Res-21-018), Drosophila (GMAC Ref No.:Res17-204), and rabbits (IACUC-TLL-24-013) were performed in accordance with the guidelines set up and approved by the Institutional Animal Care and Use Committee (IACUC) of Temasek Life Sciences Laboratory, Singapore, and adhered to Singapore’s national Bioethics Advisory Committee (BAC) and National Advisory Committee for Laboratory Animal Research (NACLAR) guidelines.

Blood from rabbit and efforts to alleviate suffering: 1) rabbit was sedated with Acepromazine at a dose of 1 mg/kg IM or SC, approximately 15 min prior to collection. 2) After blood withdrawing, apply pressure to the site for at least 2 min or until the bleeding stops. Only release the rabbit into the cage when bleeding has completely stopped. 3) The time interval between blood collection for each rabbit will be at least 2–4 weeks and the volume of blood collected was limited to 10–15 mL per collection.

Methods of sacrifice: At the end of the experimental period, rabbits were anesthetized via intramuscular injection of ketamine (30 mg/kg) and xylazine (5 mg/kg) prior to euthanasia. Once deep anaesthesia had been confirmed, rabbits were euthanized by intravenous injection of pentobarbital (150 mg/kg body weight till effect), in accordance with guidelines (IACUC-TLL-24-013).

### Key resources table

**Table:** 

REAGENT or RESOURCE	SOURCE	IDENTIFIER
**Antibodies**		
Mouse anti-FLAG M2 monoclonal antibody	Sigma-Aldrich	Cat# F1804; RRID: AB_262044
Rabbit anti-FLAG polyclonal antibody	Sigma-Aldrich	Cat# F7425, RRID:AB_439687
Mouse Myc-Tag (9B11) mAb	Cell Signaling Technology	Cat# 2276, RRID:AB_331783
Rabbit anti-GFP polyclonal antibody	Temasek Lifesciences Lab	N/A
Chicken anti-GFP polyclonal antibody	Abcam	Cat# ab101863, RRID:AB_10710875
Rabbit Anti-Myc-Tag Monoclonal Antibody	Cell Signaling Technology	Cat# 2278, RRID:AB_490778
Mouse V5 Tag Monoclonal Antibody	Thermo Fisher Scientific	Cat# R960-25, RRID:AB_2556564
Mouse histone 2A gamma variant, phosphorylated antibody (p-γ-H2Av)	DSHB	Cat# UNC93-5.2.1, RRID:AB_2618077
Rabbit Histone H2AvD phosphoS137 Antibody	Rockland	Cat# 600–401-914S, RRID:AB_11183655
Mouse Fasciclin III (Drosophila) antibody	DSHB	Cat# 7G10, RRID:AB_528238
Mouse anti-α-Tubulin monoclonal antibody	Sigma-Aldrich	Cat# T9026, RRID:AB_477593
Mouse anti-Histone Antibody, clone H11-4	Sigma-Aldrich	Cat# MAB3422
Rabbit anti-acetyl-Histone H4 Antibody	Sigma-Aldrich	Cat# 06-866
EZview Red Protein G Affinity Gel	Sigma-Aldrich	Cat# E3403-1ML
Cy3 AffiniPure Goat Anti-Mouse IgG (H + L)	Jackson ImmunoResearch	Cat#**115-165-003** RRID: AB_2338680
Alexa Fluor 488 AffiniPure Donkey Anti-Mouse IgG (H + L)	Jackson ImmunoResearch	Cat#**715-545-150** RRID: AB_2340846
Cy3 AffiniPure Goat Anti-Rabbit IgG (H + L)	Jackson ImmunoResearch	Cat#**111-165-003** RRID: AB_2338000
Alexa Fluor 488 AffiniPure Goat Anti-Rabbit IgG (H + L)	Jackson ImmunoResearch	Cat#**111-545-003** RRID: AB_2338046
Alexa Fluor 647 AffiniPure Goat Anti-Mouse IgG (H + L)	Jackson ImmunoResearch	Cat#**115-605-146** RRID: AB_2338912
Alexa Fluor 647 AffiniPure Goat Anti-Rabbit IgG (H + L)	Jackson ImmunoResearch	Cat#**111-605-003** RRID: AB_2338072
Peroxidase AffiniPure Goat Anti-Rabbit IgG (H + L)	Jackson ImmunoResearch	Cat#**111-035-003** RRID: AB_2313567
Peroxidase AffiniPure Goat Anti-Mouse IgG (H + L)	Jackson ImmunoResearch	Cat#**115-035-003** RRID: AB_10015289
**Bacterial and Cell Strains**		
One Shot TOP10	Thermo Fisher Scientific	Cat# C404010
XL10-Gold Ultracompetent Cells	Stratagene	Cat#200314
*D. melanogaster* Cell line S2: S2-DRSC	William Chia	S2-DGRC: 181
Aedes albopictus C6/36 cells	Chua BW	ATCC, CRL-1660
Aedes aegypti Aag-2 cells	Julien Pompon	N/A
**Chemicals, Peptides, Recombinant Proteins, and Enzymes**		
Protease Inhibitor Cocktail Tablets (Complete, EDTA-free)	Sigma-Aldrich (Roche)	Cat# 000000005056489001
SuperSignal West Pico Chemiluminescent Substrate	Thermo Fisher	Cat# 34080
SuperSignal West Femto Maximum Sensitivity Substrate	Thermo Fisher	Cat# 34096
VECTASHIELD Mounting Medium	Vector Laboratories	Cat# H-1400
Blotting-Grade Blocker	Bio-Rad	Cat# 170-6404
DMEM	Thermo Fisher (Gibco)	Cat#41965-039
Trypsin-EDTA (0.25%)	Thermo Fisher	Cat# 25200056
Fetal Bovine Serum (FBS)	Thermo Fisher (Gibco)	Cat# A5209502
Shields and Sang M3 Insect Medium	Sigma-Aldrich	Cat# S3652-6X500ML
Lipofectamine 2000	Thermo Fisher	Cat# L3000015
Effectene Transfection Reagent	Qiagen	Cat# 301427
KAPA HiFi (100units)	Kapa Biosystems	Cat# KK2101
*PfuUltra* HF DNA polymerase	Agilent Technologies,	Cat#600382
**Other reagents**		
polyvinyl difluoride (PVDF) membranes	Millipore	Cat# ISEQ00010
**Experimental Models: Organisms/Strains**		
*D. melanogaster*: *w*^*1118*^	William Chia	N/A
Aedes aegypti Liverpool strain	Chun-Hong Chen and Omar S. Akbari	N/A
**Software and Algorithms**		
ImageJ/FIJI	NIH	https://imagej.net/Fiji
Prism 8	GraphPad	https://www.graphpad.com/scientific-software/prism/
Adobe Photoshop CS6	Adobe	N/A
Adobe Illustrator CS6	Adobe	N/A
Leica LAS/LAS AF lite	Leica	N/A
IMAGE LAB5.2.1	Bio-Rad	N/A
BioEdit	Ibis Therapeutics	N/A
**Recombinant DNA**		
pAC5.1C vector	Invitrogen (Thermo Fisher)	**Cat# V**411020
pENTR/D-TOPO entry vector	Invitrogen	**Catalog number K240020**
pB-OpiE2-DsRed	Eric Marios	**N/A**
Piggybac helper plasmid	Roenick Proveti Lomo and Eric Marios	**N/A**
*Topo-cifA*_*wAlbB[TIII]*_	This paper	
*Topo-cifB*_*wAlbB[TIII]*_	This paper	
*Topo-NLS-cifB*_*wAlbB[TIII]*_ *-NLS*	This paper	
*Topo-flag-cifA*_*wAlbB[TIII]*_ *-T2A-myc-cifB*_*wAlbB[TIII]*_	This paper	
*pAFW-cifA*_*wAlbB[TIII]*_	This paper	
*pAMW-cifB*_*wAlbB[TIII]*_	This paper	
*pAGW-cifB*_*wAlbB[TIII]*_	This paper	
*pAGW-NLS-cifA*_*wAlbB[TIII]*_ *-NLS*	This paper	
*pPFW-cifA*_*wAlbB[TIII]*_	This paper	
*pPMW-cifB*_*wAlbB[TIII]*_	This paper	
*pPMW-NLS-cifB*_*wAlbB[TIII]*_ *-NLS*	This paper	
*pMW-flag-cifA*_*wAlbB[TIII]*_ *-T2A-myc-cifB*_*wAlbB[TIII]*_	This paper	
*Topo-cifA*_*wPip[TI]*_	This paper	
*Topo-cifB*_*wPip[TI]*_	This paper	
*pAFW-cifA*_*wPip[TI]*_	This paper	
*pAGW-cifB*_*wPip[TI]*_	This paper	
		
		

**Table:** 

Primers for molecular cloning	
*cifA*_wAlbB[TIII]_.fp	CACCATGCCAAAAAGTAAAACTAAACG
*cifA*_wAlbB[TIII]_.revp	CTATTTGTTAACAACTGCAGCAG
*cifB*_wAlbB[TIII]_.fp	CACCATGCATGGTAATAATGAAGATCG
*cifB*_wAlbB[TIII]_.revp	TCATCTAGAAAACCCAGATGCTCTAC
*cifA*_wAlbB[TIII]_-*NLS*-f	CACCATGCCGGCAGCTAAGAAAAAGAAACTGGATCATGGTAATAATGAAGATC
*cifB*_wAlbB[TIII]_-NLSERVrevp	GGCCGCACTGTGCTGGATATCTCAGACTTTCCTCTTCTTCTTG
flag-*cifA*_wAlbB[TIII]_-T2Afp	GGGCGGCCGCATGGACTACAAAGACGATGACGACAAGCCAAAAAGTAAAACTAAACG
Flag-*cifA*_wAlbB[TIII]_-T2Arp	GGTCTAGAGGTCTCAAGGGCCGGGATTCTCCTCCACGTCACCGCATGTTAGAAGACTTCCTCTGCCCTCTTTGTTAACAACTGCAGCAG
myc*-cifB*_wAlbB[TIII]_-T2Afp	GGGGTCTCACCCTATGGAACAAAAACTTATTAGCGAAGAAGATCTTCATGGTAATAATGAAGATCG
myc*-cifB*_wAlbB[TIII]_-T2Arp	GGCTCGAGTCATCTAGAAAACCCAGATG
*cifA*_wPip[TI]_.Af	CACCATGCCAACACAGAAAGAGCTTC
*cifA*_wPip[TI]_.revp	TTACTTATTACCACTAAGCGTAAAG
*cifB*_wPip[TI]_.f	CACCATGAGTAATGGTGATGGACTTA
*cifB*_wPip[TI]_.revp	CTACCTACTACCTCTACTATTG

### Plasmids and molecular cloning

*cifs*_wAlbB[TIII]_ genes were cloned from *Aedes albopictus* mosquito, which is doubly infected with *w*AlbA and *w*AlbB [64]. *cifs*_wPip[TI]_ were cloned from *Culex quinquesfasciatus* mosquito, which is infected with *w*Pip. Briefly, adult *Aedes albopictus* mosquitoes were anesthetized by chilling on ice. After sterilized in 70% ethanol for 5 min and rinsed for 3–5 times in PBS, individual mosquitoes were dissected in PBS in one well of a 9-well glass plate (Pyrex spot plate with nine depressions, corning 7220) under a dissecting microscope (Olympus). Ovaries were transferred into 1.5 mL Eppendorf microcentrifuge tube and homogenized using a disposable polypropylene pestle. Mosquito DNA was extracted with the Phenol-Chloroform-Isoamyl alcohol method. Coding region of *cifs*_wAlbB[TIII]_ genes, *cifA*_wAlbB[TIII]_ and *cifB*_wAlbB[TIII]_, were amplified by PfuUltra HF DNA polymerase (Agilent Technologies, Catalog# 600382) and cloned into the pENTR/D-TOPO entry vector (Thermo Scientific). As a control, eGFP was cloned into the pENTR/D-TOPO entry vector. Those clones were recombined into pAFW, or pAMW, using LR Clonase Mix II (Thermo Scientific) to generate N-terminal *flag*, *myc, or GFP* tag version respectively.

To make a nuclear targeting *cifA*_wAlbB[TIII]_ construct, sequences of 2 nuclear localization signals (NLS) were added to the 5’ end and 3’ end of the coding sequence, respectively. The 5’ NLS (*CCGGCAGCTAAGAAAAAGAAACTGGAT* (amino acid: PAAKKKKLD)) and 3’ SV40 NLS (*CCCAAGAAGAAGAGGAAAGTC* (Amino acid: PKKKRKV)) were adapted from the Addgene plasmid pCSDest2-2XNLS-SpCas9-WT-NLS-3XHA-NLS (Plasmid #69232) [65].

To generate a *cifA*_wAlbB[TIII]_*-T2A-cifB*_wAlbB[TIII]_, expression cassette mimicking *Wolbachia* operon-like arrangement, a *flag* sequence was added to the 5’ end of the *cifA*_wAlbB[TIII]_ (with stop codon removed) before adding a T2A (*GAGGGCAGAGGAAGTCTGCTAACATGCGGTGACGTCGAGGAGAATCCTGGACCT,* amino acid sequence: EGRGSLLTCGDVEENPGP) sequence to its 3’ end, which was subsequently cloned into a pB vector. After sequencing confirmation, a *cifB*_wAlbB[TIII]_ with a *myc* sequence attached to its 5’ end was cloned, in frame with T2A sequence, into the vector.

PCR products of *NLS-cifA*_wAlbB[TIII]_-*NLS* and *cifA*_wAlbB[TIII]_*-T2A-cifB*_wAlbB[TIII]_ were amplified using PfuUltra HF DNA polymerase and cloned into pENTR/D-TOPO entry vector. Those TOPO vectors were recombined into destination constructs by the gateway cloning system. The following constructs were generated for making transgenic *Drosophila* stocks.

- *pPFW-cifA*_*wAlbB[TIII]*_ with N-terminal *flag* tag
- *pPMW-cifB*_*wAlbB[TIII]*_ with N-terminal *myc* tag
- *pPW-cifA*_*wAlbB[TIII]*_*-T2A-cifB*_*wAlbB[TIII]*_ with *cifA*_wAlbB[TIII]_ fused with N-terminal *flag* tag and *cifB*_wAlbB[TIII]_ with N-terminal *myc* tag
- *pPMW-myc-NLS-cifA*_*wAlbB[TIII]*_*-NLS* with N-terminal *myc* tag

After being confirmed by sequencing and testing in S2 cells, transgenic constructs were sent to BestGene for generating transgenic *Drosophila* stocks.

### Cell culture, transfection, immunofluorescence staining, and western blotting analysis

#### *Drosophila* S2 cell line.

*Drosophila* Schneider (S2 from DGRC) cell culture and transfection were described as before [66]. Briefly, S2 cells were cultured in Shields and Sang M3 Insect Medium (Sigma) supplemented with 10% FBS. Cells were grown in a 25 cm^2^ flask in a 25°C incubator and routinely passaged every 3 days.

#### Mosquito cell lines.

*Aedes albopictus* C6/36 cells (ATCC, CRL-1660) were maintained in Dulbecco’s modified Eagle medium (DMEM) (Gibco) supplemented with 10% Fetal bovine serum (FBS). C6/36 cells were cultured at 28°C in a 5% CO_2_ atmosphere. C6/36 cells were passaged every week. *Aedes aegypti Aag*-2 cells were maintained at 28°C in *Drosophila* Schneider’s medium with L-glutamine (Gibco) supplemented with 10% Fetal Bovine Serum (FBS) and were passaged every 3 days.

### Transfection, immunofluorescence staining, and imaging

All S2 cell transfections were performed using Effectene Reagent (Qiagen) according to the manufacturer’s instructions. 0.5 µg of each plasmid was transfected into S2 cells. Mosquito C6/36 or Aag2 cell transfections were performed using lipofectamine 2000 according to the manufacturer’s instructions. For immunofluorescence staining, autoclaved coverslips were put in a 6-well plate before cells were seeded. 48 h after transfection, cells were fixed in 4% paraformaldehyde in PBS for 20 min, followed by quick rinses with PBS for 3 times and permeabilized in washing buffer (PBST, 0.1% Triton-X-100 in PBS) for more than 1 hr by shaking at low speed on an orbital shaker (MS Major Science, MS-NOR-30). The fixed cells were then blocked in PBST supplemented with 5% NGS (newborn goat serum) for 30 min. After that, cells were incubated with primary antibodies in PBST with 5% NGS overnight. The following day, cells were washed with washing buffer 3–5 times before incubating with secondary antibodies conjugated to Alexa Fluor 555, 488, or 633 (Jackson ImmunoResearch or Thermo Fisher) for an additional 3 h at room temperature. DNA was stained with Hoechst 33342 (Thermo Fisher, cat. 62249). For *Drosophila* ovary or testis, tissues were dissected in Sang M3 Insect Medium (S2 cell culture medium) supplemented with 10% FBS. After fixed by 4% paraformaldehyde (PFA) in PBS for 20 min at room temperature, the staining procedure followed the steps for cultured cell staining. After incubation with a secondary antibody and washing with PBST, the samples were mounted with VECTASHIELD mounting medium (VECTOR LABORATORIES INC). Primary antibodies used in this study were: anti-Flag (1:2000, Sigma, F1804 mouse), anti-Myc (1:1000, rabbit), anti-V5 (1:500, mouse), anti-Fasciclin III (1:5000, mouse), anti-GFP (1:5000, chicken), anti-p-H2AvD (1:5000, rabbit), and anti-p-γ-H2Av (1:2000, mouse). Images were taken using a Leica SPE8 confocal microscope and processed with Adobe Photoshop.

### Transfection, immunoprecipitation and Western Blot Analysis

To detect protein interaction, plasmids were transfected into S2 cells in 6-well plate. 2 days later, cells were harvested and lysed with 250 µL of Nonidet P-40 lysis buffer (50 mm Tris, pH 8.0, 150 mM NaCl, 0.5% Nonidet P-40, 10 mM NaF, 0.2 mM EGTA, protease inhibitor cocktail (Roche)) and rocked occasionally on ice for 30 min. The lysate was collected and cleared by centrifugation at 13,000 rpm for 5 min at 4 °C. Cell lysates were mixed with anti-GFP antibody (produced in the lab) at 4 °C for 2 h with shaking, then 30 μL of 1:1 slurry of EZview Red Protein G Affinity Gel (Sigma, E3403) was added to the mixture and incubated overnight. Protein G beads were sedimented by centrifugation (1 min, 8000 rpm, 4 °C), washed 3–5 times with lysate buffer, and resuspended in 25 μL of 2 × SDS sample buffer. After boiling, proteins were separated by 10% SDS-polyacrylamide gel electrophoresis and transferred onto polyvinylidene difluoride membranes (ImmobilonTM-P, Millipore). Membranes were blocked with 5% non-fat milk (Blotting-Grade Blocker, Bio-Rad, cat# 170–3404) in TBST buffer (10 mm Tris/HCl (pH 7.5), 150 mm NaCl, 0.1% (v/v) Tween 20) for 1 h at room temperature and incubated with first antibody in TBST plus 3% BSA at 4 °C for overnight. After five washes with TBST buffer, membranes were incubated with horseradish peroxidase-conjugated second antibody diluted 1:10,000 in 5% non-fat milk in TBST buffer. After washing with TBST buffer, chemoluminescence detection was performed by exposure of SuperSignal West Pico Chemiluminescent Substrate (Thermo Fisher)-treated membranes ChemiDoc (touch screen model, 2017, Bio-Rad). Data analysis by IMAGE LAB5.2.1and photoshop software.

### Drosophila genetics

The following lines were used in this paper. Detailed information for the fly strains used is described in Flybase (flybase.bio.indiana.edu) except where noted. Fly stocks were obtained from Bloomington Drosophila Stock Center (BDSC. Following strains and stocks were used: *w*^*1118*^, *nos-gal4*, *bam-gal4*, *NTG40-gal4* (from Pei Jun-Wei lab), *brat-gal4*, *rbp4-gal4*, and *topi-gal4*. Those tested *gal4* were list in Table 1. Transgenic stocks including pUASp-*flag*-*cifA*_wAlbB[TIII]_, pUASp-*myc-cifB*_wAlbB[TIII]_, pUASp-*myc*-*NLS-cifB*_wAlbB[TIII]_-*NLS*, pUASp-*flag*-*cifA*_wAlbB[TIII]_*-T2A-myc-cifB*_wAlbB[TIII]_ were injected and established by BestGene, via standard P-element mediated transformation. The transgenic stocks *yw*; pUASp-6His-V5-CidA-T2A-Flag-CidB-attP/ + was kindly provided by J.F. Beckmann via Benjamin Loppin and Frederic Landmann. All strains were maintained at 25^o^C.

### Mosquito breeding

*Aedes aegypti* mosquitoes were reared in the environment chamber with consistent growing conditions (28°C, 80% relative humidity, 12-h photoperiod) throughout their development according to the methods as described by Zhang et al [67]. In brief, eggs were hatched in the hatching buffer (Tryptone: Sodium Chloride: Yeast Extract; ratio 2:2:1) and vacuumed for 15 min to ensure consistent age of the cohort. Four hundred first instar larvae were manually counted and transferred to a plastic container that contained 500 mL of sterile water. The larvae were fed with 50mg, 64mg, 130mg, 260mg, and 400mg of larval food on days 1–5, respectively. Larval food is a mixture of yeast and ground Tetramin Fish Flakes in a ratio of 1:1. The larvae were fed with a diet containing yeast only on days 1 and 2. The pupae from the breeding bowl were sorted out and placed in a mosquito cage (Bugdorm, Taiwan; Dimension: W24.5 x D24.5 x H24.5 cm) to allow for eclosion. Two small vials with cotton soaked in water and 10% sugar solution with Vitamin B complex as a supplement were placed in the cage. Mature (day 3–5 post-emergence) adult mosquitoes were fed with rabbit blood using an artificial membrane feeding system (Hemotek Ltd., UK or Orinno Technology, Singapore) to produce eggs. Egg paper was placed two days post-blood feeding for egg laying and was dried after another two days for storage.

### Mosquito embryo microinjection

Three days post blood meal, female mosquitoes were transferred into a large Drosophila vial with filter paper and wet cotton at the bottom for egg collection in the dark. After 40–50 min egg laying, mosquitoes were removed, and the filter paper was checked under a microscope to ensure that enough eggs were collected. Eggs in light gray colour were aligned in line on another wet filter paper, and the posterior end (thin side) of the eggs were placed in the same direction. Once 100 eggs were aligned, the filter paper was dried with tissues as much as possible, which was important for egg transfer at the next step. A cover slip with double-sided tape on one side was prepared. By gently pressing the eggs with the cover slip at the tape side, the eggs were stuck to the tape. The eggs were then covered with halocarbon 700 oil. A 10 µL injection mix was prepared for the piggyBac system: 100 ng/µL plasmid, 50 ng/µL piggyBac helper, 1 µL injection buffer (10x stock: 5 mM KCl, 0.1 mM sodium phosphate), and topped up with water to 10 µL. Eggs were injected with a small volume of injection mix at the posterior end with a microinjector (Eppendorf). After injection, eggs were aligned back to a wet filter paper placed in a beaker and transferred to an incubator with 80% relative humidity and 28°C for three days for maturation. The injected eggs were hatched, and positive larvae were screened under a fluorescent microscope (Leica Fluorescence Stereoscope M205 FCA) with the selection marker. Once pupa formation was complete, male and female pupae were separated based on pupa size (males were smaller than females) and were crossed with WT females and males, respectively. G_1_ eggs were hatched, larvae were screened, and adults were crossed as following the procedure mentioned above. Repeat this crossing scheme until acquiring larvae with a single insertion (50% positive ratio and consistent selection marker intensity).

### Embryo collection and fixing

Around 150–200 *Drosophila* virgin females were crossed with an equal number of males in a bottle and were fattened for two days before egg collection. The flies were transferred into an empty bottle capped by a gel plate to allow egg laying. Yeast paste was placed at the center of the gel plate, and the bottle was settled upside down to start egg collection. Embryos were collected within 30 min, washed off with PBT, and transferred to a metal net with an artistic brush. 50% bleach solution was used to dechorionate the eggs for 3 min, and the eggs were flushed with tap water and immediately dried on tissue paper. The metal shell was then transferred into a scintillation vial containing 5 mL heptane and 5 mL freshly made fixation solution (4% PFA and 0.1% HEPES in PBS). Gently shake or vortex the vial for 12 min and discard the fixation solution (bottom layer) using a glass Pasteur pipette. 5 mL of methanol was added to shake for 1 min, and the vial was put on the bench for several minutes to let the embryos settle to the bottom. The embryos were collected into a 1.5 mL Eppendorf tube and were washed three times with ethanol. Eggs are stored in ethanol at -20 °C or proceed to the immunostaining.

### Immunostaining and confocal imaging

*Mosquito* or *Drosophila* tissue samples were dissected in PBS in case of further indication. Samples were then fixed for 20 min in an Eppendorf tube (pre-coated with 3% BSA) with fixation solution (4% PFA and 0.1% HEPES in PBS). Fixation solution was then discarded, and samples were washed three times with PBT (0.1% Triton in PBS), 15 min each time. Samples were blocked with 3% BSA in PBT for at least 30 min and incubated with the primary antibodies overnight at room temperature. After three times washing with PBT, samples were incubated with secondary antibodies and DNA dye (Hoechst) for two hours in the dark. Samples were then washed three times, and mounting medium was added to prevent photobleaching. Samples were stored at -20 °C or mounted on a glass slide for confocal imaging (Leica SP8 confocal system).

### *Wolbachia* transinfection and subsequent cross

Approximately ten blood-fed *Ae. aegypti* females and ten blood-fed *Ae. albopictus* females were held in vials for 1-h oviposition, respectively. Gray eggs from both strains were aligned on a double-sided tape covered with halocarbon 700 oil. Cytoplasm from *Ae. albopictus* donor eggs were withdrawn and transferred into the posterior end of *Ae. aegypti* recipient eggs through an injection needle (aluminosilicate glass capillary tube, O.D.: 1.0mm, I.D.: 0.64mm, Sutter Instrument) with a microinjector. After injection, eggs were aligned back on a wet filter paper and transferred to incubator at 80% relative humidity and 28°C for three days for maturation. Injected (G_0_) eggs were hatched subsequently and reared as described above.

G_0_ virgin females were crossed with WT *Ae. aegypti* lab strain. After oviposition, G_0_ females were screened by PCR to check the existence of *Wolbachia*. G_1_ eggs from *Wolbachia*-positive G_0_ females were hatched, and G_1_ females were crossed again with the WT strain and blood fed, individualized, and allowed for oviposition, followed by PCR screening. Offspring from positive G_1_ females were self-crossed for another six generations to stabilize *Wolbachia* inheritance. Stable strain was subjected to sample collection for proteomics analysis.

### Shotgun proteomics and data processing of mosquito testis and embryos

S-TRAP lysis buffer (50 mM triethylammonium bicarbonate, 5% SDS) was added to each sample and the protein lysates were processed using the S-TRAP Micro column (Protifi), according to the manufacturer’s recommendations. Samples were analysed via data-dependent acquisition (DDA) using an EvoSep One System coupled to an SCIEX TripleTOF 6600 mass spectrometer. For each sample, 1 μg of peptides was loaded onto each Evotip, following the manufacturer’s protocol. The IonOpticks Aurora Elite SX 1.7 μm C18 column (75 μm x 150 mm) was used as the analytical column. The mobile phases used were 0.1% formic acid in water (Solvent A) and 0.1% formic acid in acetonitrile (Solvent B). The Whisper 40 SPD method was used. The ionization parameters used were 8 units for GS1, 30 units for curtain gas, 1800 V for IonSpray voltage floating, 150°C for interface heater temperature. The precursor ion scan was done in the range of 400–1600 m/z with 250 ms accumulation time, in the positive ion mode. 50 precursors with charge state 2–5 and intensity > 150 cps were selected for fragmentation. Dynamic exclusion was applied for 15 seconds. For each precursor selected, a MS/MS scan was done for the mass range of 100–1800 m/z with 50 ms accumulation time in high sensitivity mode.

Data processing was done using PEAKS Studio 11.5, using UniProt *Aedes aegypti* reference proteome (UP000008820, 2024_01 release, 20640 entries), UniParc *Wolbachia pipientis w*AlbB (*w*AlbB) proteome (UP000290937, 2024_01 release, 1022 entries), spiked with common contaminant proteins. The precursor and fragment error tolerance used are 15 ppm and 0.05 Da, respectively. The digestion enzyme was set to trypsin, with a maximum of 3 missed cleavages. The fixed modification on cysteine from alkylation was beta-methylthiolation, and the variable modifications were acetylation (N-term) and oxidation (M) with a maximum of 3 variable PTM per peptide. The deep learning boost was used. Reported proteins were filtered at 1% false discovery rate (FDR).

The mass spectrometry proteomics data have been deposited to the ProteomeXchange Consortium via the PRIDE [68] partner repository with the dataset identifier PXD084067.

### *Drosophila* testis dissection and western blotting analysis

Drosophila testis tissues were dissected in Sang M3 Insect Medium (S2 cell culture medium). As an experimental plan, the testis was cut at a 1/4-–1/3 position from the tip, as shown in Fig 4D. All testis head parts (the anterior portion) and tail (the remaining posterior portion) parts were collected into Eppendorf tubes, respectively. Liquid was removed as much as possible. Relevant lysis buffer (Nonidet P-40 lysis buffer containing 50 mM Tris, pH 8.0, 150 mM NaCl, 0.5% Nonidet P-40, and 0.2 mM EDTA with protease inhibitor cocktail (complete, Roche)) was added to a final volume of one microliter/4 pairs of testes. Tissue was homogenized using a disposable polypropylene pestle. 6X protein loading buffer (Laemmli buffer) was added. After boiling, proteins were separated by 10% SDS-polyacrylamide gel electrophoresis and transferred onto polyvinylidene difluoride membranes (Immobilon-P, Millipore). Membranes were blocked with 5% non-fat milk (Blotting-Grade Blocker, Bio-Rad, cat# 170-3404) in TBST buffer (10 mM Tris/HCl, pH 7.5, 150 mM NaCl, 0.1% (v/v) Tween 20) for 1 h at room temperature and incubated with primary antibody in TBST plus 3% BSA at 4°C overnight. The following day, the membrane was washed five times with TBST buffer, then incubated with horseradish peroxidase-conjugated secondary antibody diluted 1:10,000 in 5% non-fat milk in TBST buffer. After washing with TBST buffer, chemoluminescence detection was performed by exposure of SuperSignal West Pico Chemiluminescent Substrate (Thermo Fisher)-treated membranes to Biomax MR films (Kodak) or ChemiDoc (touch screen model, Bio-Rad). Data analysis by IMAGE LAB5.2.1, photoshop and ImageJ software.

### *Drosophila* hatch rate assays

Hatch rate was performed as described previously [21]. In brief, parental males were generated by crossing *nos-gal4* virgin females with either *w*^*1118*^ (control group) or corresponding transgenes (experimental group). 0–48 h old males were collected and used for crossing. Virgin females (*w*^*1118*^) were aged at 2–5 days before crossing. For the rescue experiment, virgin females were collected from the cross of *nos-gal4* with pUASp-*flag*-*cifA*_wAlbB[TIII]_.

Before crossing, grape juice agar vials were prepared. 2.5-3 ml of grape juice agar was put into a small Drosophila stock vial (8-ounce, round-bottom, polypropylene). After cooling down to room temperature, grape juice agar vials were kept at 4^o^C room up to one month. A single pair of male and female was crossed in a grape juice agar vial with a small amount of wet yeast paste smeared on the agar. The flies were incubated at 25°C for 24–30 h, and the vials were replaced with a fresh grape juice agar vial with a small amount of yeast paste. The number of embryos on each vial was counted and stored at 25°C for 36 h. The number of unhatched embryos was counted. The percent of embryos hatched into larvae was calculated by dividing the number of hatched embryos by the total number and multiplying by 100.

To prepare grape-juice agar vials and plates, 12.5 g of agar was mixed in 350 mL of de-ionized water and autoclaved. In a separate tube, 10 mL of ethanol was used to dissolve 0.25 g Tegosept (methyl 4-hyrdoxybenzoate). After melting agar and cooling down to 60^o^C, 1.5 g of sucrose, 140mL of grape juice, and 10 mL of Tegosept were added. Mixed completely and poured into a small Drosophila stock vial or 60  × 10-mm culture dishes.

### Statistical data analysis

Statistics were calculated, and figures were generated with Graphpad Prism 8. The statistical significance of the differences was determined by multiple comparisons based on a Kruskal-Wallis test and Dunn multiple test correction. Statistical details were described in the Figure Legends. *p < 0.05, **p < 0.01, ***p < 0.001, ****p < 0.0001.

## Supporting information

S1 Fig Cifs_wPip[TI]_ localization in *S2* cells and Cifs_wAlbB[TIII]_ localization in *Aag2* cells. A. Schematic diagram of *cifA*_wPip_ and *cifB*_wPip_ constructs. B. Representative images showing Cifs_wPip[TI]_ subcellular localization and p-γ-H2Av antibody expression in *S2* cells. C. Representative images showing Cifs_wAlbB[TIII]_ subcellular localization in *Aag2* cells. Scale bar, 5 µm.
(TIF)

S2 Fig Overall expression patterns of different germline gal4 lines in Drosophila testis. Expression pattern of nos-gal4, *NGT40-gal4*, *bam-gal4*, *brat-gal4*, *rbp4-gal4* and *topi-gal4* when crossing with *UAS-mCD8-eGFP* responder line.
(TIF)

S3 Fig Differential germline expression of Cifs_wAlbB[TIII]_ and Cifs_wPip[TI]._ A. CifA_wPip[TI]_ and CifB_wPip[TI]_ are detected in late spermatogonia, spermatocytes and spermatids when ectopically expressed in the fly testes by *brat-gal4* or *topi-gal4* driver. FasIII indicates the hub cells (dot circle). B. Both CifA_wAlbB[TIII]_ and CifB_wAlbB[TIII]_ are weakly detectable in late spermatogonia and early spermatocytes but not late spermatocytes and spermatids when ectopic expression by spermatogonia driver *brat-gal4*. Both CifA_wAlbB[TIII]_ and CifB_wAlbB[TIII]_ are not detected in spermatocytes and spermatids when expressed by spermatocyte driver *rbp4-gal4* and *topi-gal4*. C, In *Aedes aegypti*, CifA_wAlbB[TIII]_ and CifB_wAlbB[TIII]_ are not detected when ectopically expressed under the strong spermatocyte-specific β2t promoter. Left panels are the overview. Right panels indicate late spermatocyte/spermatid stage.
(TIF)

S1 Table List of *Wolbachia* proteins detected in *w*AlbB-carrying mosquito testis in all three replicates. (XLSX)

S1 File Rawgel. (TIF)

S2 File Fig 2C raw. (XLSX)

S3 File Fig 2E raw. (XLSX)

S4 File Fig 2F raw. (XLSX)

S5 File Fig 3B raw. (XLSX)

S6 File Fig 3C raw. (XLSX)

S7 File Fig 3E raw. (XLSX)
